# Structural and Functional Enhancement of Halal Gelatin Capsules Reinforced with Corn Husk Cellulose

**DOI:** 10.3390/polym17202803

**Published:** 2025-10-21

**Authors:** Flora Elvistia Firdaus, Aurelia Kinanti

**Affiliations:** Department of Chemical Engineering, Jayabaya University, Jl. Pulomas Selatan Kav 23, Jakarta 13210, Indonesia; aurelliakinanti13@gmail.com

**Keywords:** corn husk cellulose, halal gelatin capsules, biopolymer reinforcement, tensile strength, disintegration behavior, FTIR analysis

## Abstract

Corn husk-derived cellulose (CHC) was incorporated into gelatin–cassava starch (CS) capsule formulations to evaluate its effectiveness as a sustainable reinforcing agent. The addition of CHC enhanced the structural cohesion of the films and improved their resistance to storage-related temperature–humidity stress, while maintaining desirable flexibility. Consistent with this, the films retained mechanical performance and appearance under ICH-aligned storage conditions, indicating better endurance during storage and processing. Disintegration performance remained within pharmacopeial requirements in both acidic and neutral media, confirming the suitability of the capsules for oral delivery applications. Surface assessment revealed more uniform morphology and fewer irregularities in the capsule matrix when CHC was present, suggesting strong compatibility among the cellulose, gelatin, and starch components. Collectively, these findings demonstrate that CHC is an effective plant-based reinforcement capable of strengthening gelatin capsules without compromising functional performance. The use of corn husk, an abundant agricultural residue, also highlights a sustainable pathway for the development of halal-compliant capsule shells and contributes to the broader advancement of eco-friendly biopolymer systems in pharmaceutical applications.

## 1. Introduction

Corn (*Zea mays* L.) is a staple crop that provides carbohydrates, proteins, lipids, and essential minerals, making it a vital global food and feed source. Its kernels contain 70–80% starch, 8–10% protein, and minor fractions of oil and fiber, whereas its husks—typically treated as waste—are composed primarily of cellulose, hemicellulose, and lignin [1,2]. This dual composition highlights corn not only as a nutritional commodity but also as a promising raw material for sustainable biopolymer applications. Among reinforcing agents, corn husk cellulose (CHC) is attractive due to its strength, biodegradability, and compatibility with polymer matrices; however, its hydrophilic character can lead to moisture sensitivity [3,4].

Gelatin remains the most widely applied capsule material; porcine gelatin provides superior mechanical stability but is unsuitable for halal use, while bovine gelatin is halal-compliant yet exhibits relatively lower strength and thermal resistance [5,6]. Cassava starch (CS) represents another renewable polysaccharide that enhances compatibility and degradability; however, its brittleness and water sensitivity limit its direct use [7]. In this regard, CS is particularly relevant: abundant in tropical regions, rich in amylopectin, and widely applied in biodegradable packaging and pharmaceutical formulations [8,9].

Despite extensive studies on gelatin reinforcement, the integration of CHC with CS in bovine gelatin capsules remains underexplored. Addressing this gap offers opportunities to improve the mechanical and functional performance of halal capsules while advancing circular bioeconomy principles. Accordingly, this study develops and evaluates CHC-reinforced capsule films, with a focus on structural integrity, stability, and pharmacopeial compliance for drug delivery applications. While cellulose from other agricultural residues has been investigated for polymer reinforcement, its integration with CS in halal-compliant bovine gelatin capsules has not been reported to date [10,11,12]. This study, therefore, introduces a novel biocomposite system that simultaneously valorizes corn husk waste, leverages CS as a tropical bioresource, and addresses the mechanical limitations of bovine gelatin. The proposed approach not only advances capsule performance but also aligns with sustainability and halal requirements, offering a distinctive contribution beyond existing gelatin reinforcement strategies.

Accordingly, this study develops and evaluates CHC-reinforced gelatin–CS films, focusing on structural integrity, stability, and pharmacopeial compliance for drug delivery applications. While cellulose from other agricultural residues has been studied as polymer reinforcement, its combination with CS in halal-compliant bovine gelatin capsules has not been reported to date. This integration establishes the novelty of the present work: a dual-bioresource reinforcement system that enhances mechanical performance, ensures halal compliance, and advances circular bioeconomy principles.

Given these considerations, we hypothesize that CHC reinforcement can strengthen bovine gelatin–CS capsules through hydrogen-bonding interactions that restrict chain mobility and enhance cohesion. Specifically, CHC incorporation is expected to (i) improve tensile strength and modulus retention under stressed conditions of Relative Humidity (RH); (30 °C/75% RH, International Council for Harmonisation of Technical Requirements for Pharmaceuticals for Human Use (ICH)-aligned), (ii) maintain disintegration performance within pharmacopeial limits (≤30 min in water and 0.1 N HCl), and (iii) yield FTIR signatures indicative of CHC–gelatin–CS interactions (O–H, amide I/II, and C–O bands). Accordingly, this study systematically develops and evaluates CHC-reinforced gelatin–CS films, focusing on structural integrity, stability, and pharmacopeial compliance for drug delivery applications.

## 2. Materials and Methods

Corn husks (*Zea mays L*.) were collected from a local agricultural field in Cimanggis-Depok, Indonesia. Bovine gelatin (type B, 220 Bloom strength, food grade, (Naturlife Bogor, Bogor, Indonesia) and glycerol (≥99.5%, analytical grade, Harum Kimia were used as received. Cassava starch (Gunung Agung, Lampung, Indonesia) was employed as a polysaccharide component

- *Pretreatment of Corn Husks*

Corn husks were first thoroughly washed with distilled water to remove dust and adhering impurities, then oven-dried at 60 °C for 24 h. The husks were washed with distilled water, oven-dried at 60 °C for 24 h, and manually cut into ~1 × 1 cm^2^ pieces. Size reduction was performed using a mechanical grinder followed by sieving to obtain powder fractions of <250 µm (measured with sieve mesh ASTM E11). Alkaline pretreatment was performed by suspending 10 g of husk powder in 200 mL of 5% (*w*/*v*) NaOH solution, followed by heating at 80 °C for 2 h with continuous stirring. The slurry was filtered and washed repeatedly with distilled water until a neutral pH was reached. The alkali-treated fibers were subjected to bleaching in a 1.7% NaClO_2_ solution at 70°C for 1 h. After bleaching, the fibers were rinsed with distilled water to remove residual chemicals, oven-dried at 60 °C, and ground to yield purified cellulose powder.

- *Film preparation*

Capsule films were prepared by blending gelatin, starch, cellulose, and glycerol according to the formulations listed in Table 1. Gelatin was first dissolved in distilled water at 50–70 °C under continuous stirring until a clear solution was obtained. CS and CHC were then gradually dispersed into the gelatin solution, followed by the addition of glycerol as a plasticizer. The mixture was homogenized for approximately 2 min to produce a uniform viscous gel. The gel was then cast onto flat plastic plates and left to dry at ambient temperature until film formation was complete. The dried films were carefully peeled off and conditioned at 25 °C and 60% relative humidity (RH) for at least 24 h before characterization. Percentages in Table 1 are calculated on a total-solids basis (gelatin + CS + CHC), with glycerol treated as a plasticizer and water as a solvent

### 2.1. Mechanical Properties

Mechanical performance of the capsule films was evaluated through tensile strength, modulus, and elongation at break, measured according to ASTM D882 and ISO 527-3. In parallel, film strips were conditioned under two storage conditions (25 °C/60% RH and 30 °C/75% RH) before tensile testing. Their appearance was examined using optical microscopy and visual inspection, with observations including surface uniformity, clarity, and macroscopic integrity. These qualitative assessments complement the mechanical data, providing a broader view of film stability under practical storage stresses. These data are presented together with tensile results to reflect capsule performance under environmental stress. Classifications were adapted from literature values on gelatin and biopolymer capsule films: tensile strength >40 MPa = strong, 20–40 MPa = moderate, <20 MPa = weak; elongation at break >200% = high flexibility, 50–200% = moderate, <50% = brittle. This classification reflects functional requirements for capsule handling and oral administration.

Storage conditions of 25 °C/60% RH and 30 °C/75% RH were selected based on ICH (International Council for Harmonisation) guidelines, which define 25 °C/60% RH as standard long-term storage and 30 °C/75% RH as accelerated stability testing for pharmaceutical products. These conditions simulate real-world storage and distribution environments in tropical regions, providing relevant stress factors for capsule performance evaluation.

#### Tensile Strength (MPa) and Elongation at Break (%)

Specimens of thin plastic films (10 × 50 mm; thickness measured at five random spots) were cut and conditioned at the specified relative humidity. Mechanical properties were measured using a universal testing machine at a crosshead speed of 10 mm/min. Tensile strength, modulus, and elongation at break were determined in accordance with ASTM D882, and were tested at 25 °C/60% RH and 30 °C/75% RH.

The tensile strength was calculated as the maximum force (F, N) at break divided by the initial cross-sectional area (A, mm^2^):

(1) $$\mathrm{Tensile}\;\mathrm{Strength}\;(\mathrm{MPa})\;=\;\frac{F}{A}$$

The elongation at break was reported as strain (%) at rupture, calculated as the ratio of the extended length at break (Lb) minus the original gauge length (L_0_):

(2) $$\mathrm{Elongation}\;\mathrm{at}\;\mathrm{Break}\;(\%)\;=\;\frac{Lb-L0}{L0}\times 100$$

where L_0_ is the initial length of the strip and Lb is the length at break.

### 2.2. Storage and Morphological Appearance

#### 2.2.1. Capsule Surface by Optical Microscopy

Surface morphology was assessed using optical microscopy, which allowed for evaluation of film clarity, visible texture, and homogeneity. These observations are reported as storage appearance and are intended to complement mechanical and disintegration results. SEM analysis was not pursued, as optical-level assessment provided sufficient correlation with functional properties (mechanical strength, disintegration, and swelling), which are the focus of capsule performance evaluation. In addition to mechanical testing, films were conditioned at 25 °C/60% RH and 30 °C/75% RH to simulate storage stress. Observations of appearance and the retention of tensile properties under these conditions were collectively described as stability under storage stress.

Film samples (≈1 cm^2^) were examined by optical microscopy at 40×–100× magnification under bright-field illumination. For each formulation, three fields were imaged and qualitatively evaluated for surface uniformity, visible texture, and macroscopic defects (e.g., voids, bubbles, or aggregates). Images were captured with scale bars (100 µm) after conditioning at 25 °C/60% RH and 30 °C/75% RH (ICH).

**Figure 1:**
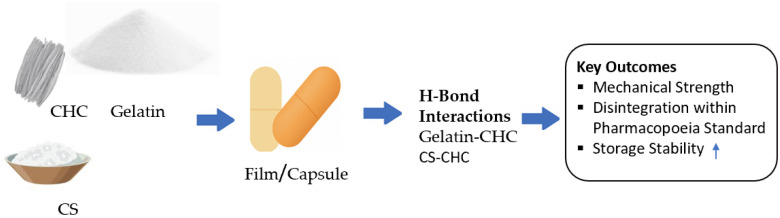
Graphical abstract of CHC-reinforced gelatin capsules showing hydrogen-bond interactions and improved performance outcomes.

Graphical abstract in Figure 1, illustrating the incorporation of CHC into gelatin–CS. Hydrogen-bond interactions between cellulose and the biopolymer matrix improve cohesion, leading to enhanced mechanical strength, compliance with pharmacopeial disintegration standards, and greater stability under storage conditions. This highlights CHC as a sustainable reinforcement pathway for halal capsule development.

#### 2.2.2. Temperature Humidity Stability Test

Physical stability was assessed under controlled storage conditions (25 °C/60% RH and 30 °C/75% RH) by monitoring changes in texture, deformation, and disintegration over 7–30 days. Film strips were conditioned at 25 °C/60% RH and 30 °C/75% RH before tensile testing (ASTM D882). In addition to mechanical properties, film appearance (clarity, uniformity, surface integrity) was observed to qualitatively assess stability under storage conditions. These data are presented together with tensile results to reflect capsule performance under environmental stress.

Film samples (2 × 1 cm) were stored under controlled conditions at 25 °C/60% RH and 30 °C/75% RH. Physical changes, including texture, deformation, and signs of disintegration, were visually examined and recorded after 7, 14, and 30 days of storage.

As these observations are macroscopic and paired with mechanical data, they primarily indicate stability under storage conditions rather than thermal transitions in the calorimetric/thermogravimetric sense. For this reason, we avoid the term thermal analysis and do not present DSC/TGA claims. Future work will incorporate DSC/TGA to quantify glass transition, melting, and degradation onset, providing complementary thermal validation.

### 2.3. Disintegration Test

Disintegration of capsule films/caps (cut to equivalent shell mass) was assessed in 0.1 N HCl (pH ≈ 1.2) using a pharmacopoeial basket-rack apparatus at 37 ± 0.5 °C, as commonly employed for simulated gastric fluid without enzymes. The acidic medium models initial gastric exposure; intactness in acid, while meeting pharmacopeial limits (≤30 min), is required before intestinal transit. Capsule film pieces (2 × 1 cm) were tested using a USP-standard disintegration apparatus. Each sample (n = 6) was placed in 700 mL of either distilled water or 0.1 N HCl, maintained at 37 ± 0.5 °C. The disintegration time was recorded as the point at which the film completely broke apart and dispersed in the medium.

### 2.4. Fourier-Transform Infrared Spectroscopy

FTIR analysis was carried out to confirm functional group interactions in the capsule films. The spectra (4000–600 cm^−1^) revealed band shifts and intensity changes consistent with hydrogen bonding and network formation among gelatin, starch, and cellulose. These spectral features support the compatibility of cellulose within the gelatin–starch matrix.

### 2.5. Film Thickness and Moisture Content (Basic Physicals)

Film thickness was measured using a digital micrometer (±0.01 mm accuracy) at five random positions on each sample, and the average value was recorded. The moisture content was determined gravimetrically by drying pre-weighed films in a hot-air oven at 105 °C until a constant weight was achieved. Moisture (%) was calculated as:

(3) $$\mathrm{Moisture}\;(\%)\;=\;\frac{Wi-Wd}{Wd}\times 100$$

where wi is the initial sample weight and wd is the oven-dried weight. All measurements were performed in triplicate, and the results are presented as mean ± standard deviation (SD).

### 2.6. Swelling Index (Water Uptake Behavior)

Phosphate-buffered saline (PBS) (pH 7.4, 0.15 M) was selected to simulate the intestinal environment, thereby providing insight into capsule performance beyond gastric conditions and reflecting physiological relevance for oral delivery applications. It enables assessment of network stability and ion-screening effects relevant to capsule integrity and performance after gastric transit. Swelling behavior was examined by immersing pre-weighed dried film samples (W_1_) in PBS at 37 °C for 30–60 min. After blotting and re-weighing (W_2_), the swelling index (%) was calculated as

(4) $$\mathrm{Swelling}\;\mathrm{Index}\;(\%)=\frac{W2-W1}{W1}\times 100$$

where W_1_ is the initial dry weight and W_2_ is the swollen weight.

### 2.7. Ash Content (Inorganic Residue/Purity)

Film samples were incinerated in a muffle furnace at 550–600 °C for 4 h. After incineration, the crucibles were cooled in a desiccator to room temperature, and the residue was weighed. Ash content (%) was calculated as:

(5) $$\mathrm{Ash}\;(\%)\;=\;\frac{Weight\;of\;Ash}{Initial\;Weight}$$

## 3. Result and Discussion

### 3.1. Mechanical Properties and Storage Appearance

The mechanical performance of capsule films was evaluated over five days under two storage conditions: 25 °C/60% RH (standard) and 30 °C/75% RH (accelerated).

#### Tensile Strength and Elongation at Break

The tensile properties of capsule films were evaluated under two storage conditions: 25 °C/60% RH (standard) and 30 °C/75% RH (accelerated). At 25 °C, tensile strength decreased from 50 MPa in Sample A to 43 MPa in Sample F, equivalent to a reduction of approximately 14%.

**Figure 2:**
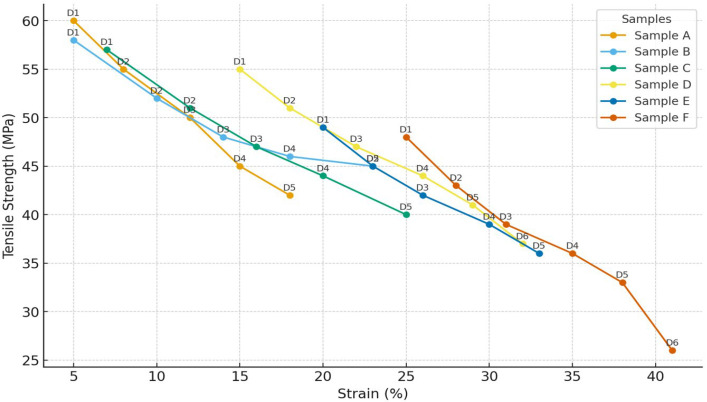
Stress–strain curves of CHC-reinforced gelatin–starch capsule films. Mechanical testing was performed at 25 °C/60% RH (long-term) and 30 °C/75% RH (accelerated), following ASTM D882. The chosen storage conditions are based on ICH stability guidelines for solid oral dosage forms, simulating real-world environments including tropical climates.

Elongation at break increased from 66.7% to 153.8% (+130%), indicating a shift toward more ductile behavior with increasing glycerol concentration. At 25 °C, the capsule films displayed a clear trade-off between rigidity and flexibility. Tensile strength started at 50 MPa in Sample A and gradually declined to 43 MPa in Sample F, reflecting about a 14% reduction. This trend suggests that increasing CHC/glycerol ratios softened the matrix and reduced its load-bearing capacity. In contrast, elongation at break rose dramatically, from 66.7% to 153.8% (+130%), indicating that the films became more flexible and ductile. In other words, the materials shifted from being rigid and elastic (Sample A) to softer and more stretchable (Sample F). At 25 °C, the capsule films displayed a clear trade-off between rigidity and flexibility. Tensile strength started at 50 MPa in Sample A and gradually declined to 43 MPa in Sample F, reflecting about a 14% reduction. This trend suggests that increasing CHC/glycerol ratios softened the matrix and reduced its load-bearing capacity. In contrast, elongation at break rose dramatically, from 66.7% to 153.8% (+130%), indicating that the films became more flexible and ductile. In other words, the materials shifted from being rigid and elastic (Sample A) to softer and more stretchable (Sample F), as shown in Figure 2.

Storage at 30 °C/75% RH accelerated mechanical degradation across all films. CHC-containing matrices retained higher integrity compared to glycerol-rich systems, indicating the reinforcing role of corn husk cellulose through hydrogen bonding, which restricted chain mobility and enhanced rigidity [10,11]. Nevertheless, under elevated temperature and humidity, the reinforcement was insufficient to fully counteract plasticization.

**Figure 3:**
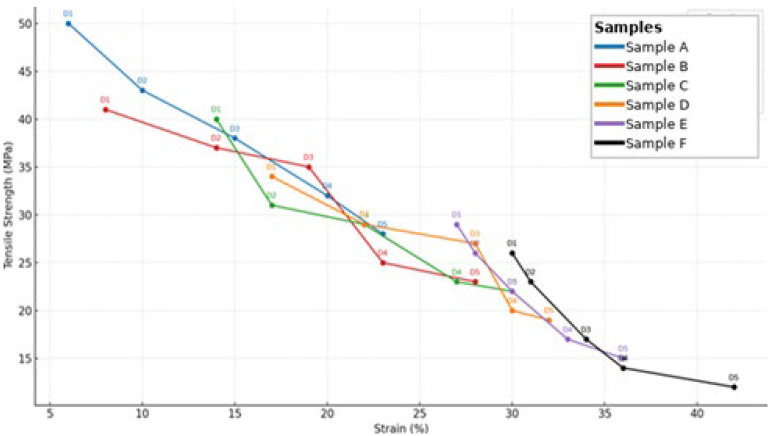
Stress–strain curves of CHC-reinforced gelatin–CS capsule films. Mechanical testing was conducted according to ASTM D882 after conditioning at 25 °C/60% RH (long-term) and 30 °C/75% RH (accelerated). These conditions follow ICH stability guidelines for solid oral dosage forms and simulate real-world storage environments, including those of tropical regions.

Tensile strength decreased from 45 MPa in Sample A to 26 MPa in Sample F (−42%). Elongation reached a maximum of 250% in Sample B (+36% relative to Sample 1) before declining in subsequent formulations, suggesting that high CHC and glycerol contents disrupted the structural network. These observations confirm that CHC contributes to improved strength and flexibility under standard conditions, but requires a balanced composition with glycerol to maintain stability under accelerated storage conditions. The graph is presented in Figure 3.

The tensile strength of the CHC-reinforced gelatin–CS films falls within (or above) the upper range typically reported for gelatin–polysaccharide composites, while preserving workable elongation for handling and forming [1,2,3]. In studies employing CHC- or CS-reinforced gelatin films, strength gains are generally attributed to hydrogen-bond-mediated chain immobilization and improved stress transfer, often at the expense of extensibility at higher filler loadings [1,2]. In line with these reports, our data show increased strength and modulus at moderate CHC contents, with a plateau or slight decline in extensibility beyond the composition corresponding to Sample B/C—consistent with network saturation effects and reduced chain mobility [2,4].

**Figure 4:**
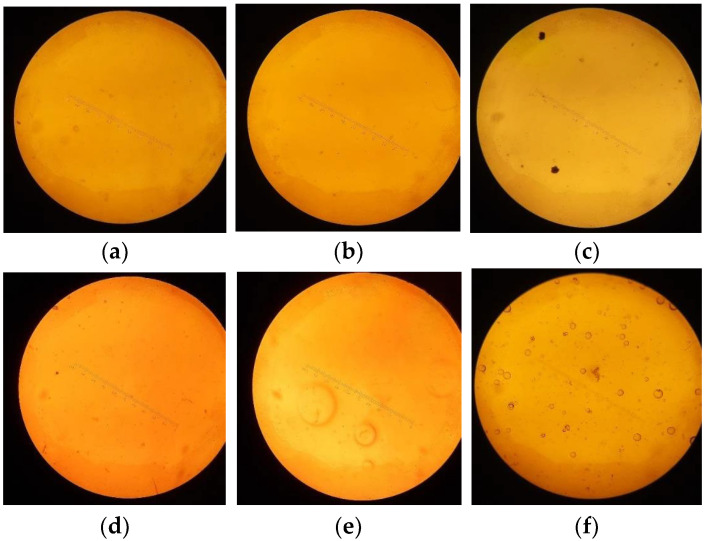
Optical micrographs (40× and 100×; scale bar = 100 µm) of capsule films: (**a**) Sample A; (**b**) Sample B; (**c**) Sample C; (**d**) Sample D; (**e**) Sample E; and (**f**) Sample F after conditioning at 25 °C/60% RH and 30 °C/75% RH (ICH). CHC-reinforced films show greater surface uniformity and clarity with fewer visible defects compared with the control.

Overall, the mechanical behavior indicates a balance between CHC and glycerol content. Films with lower cellulose content remained rigid, whereas films with higher CHC levels exhibited reduced mechanical integrity under stress. Sample B demonstrated the most stable performance, with tensile strength of 42 MPa and elongation of 250% at 30 °C. The observed tensile trends were consistent with FTIR results (Figure 4), which confirmed strong gelatin–CHC interactions, and with surface morphology analysis (Table 2), where bubble-rich structures correlated with weaker cohesion. These results confirm that the glycerol governs the mechanical stability of gelatin-based capsules–CHC ratio, highlighting the need for formulation optimization to achieve storage durability.

- Mechanical Stability of Capsule Films under Varying Storage Conditions

The use of 25 °C/60% RH and 30 °C/75% RH reflects ICH-recommended conditions for stability testing of solid oral dosage forms. The latter condition, in particular, represents high-temperature and high-humidity stress common in tropical climates, thereby enabling evaluation of capsule integrity and functionality under realistic storage scenarios.

**Figure 5:**
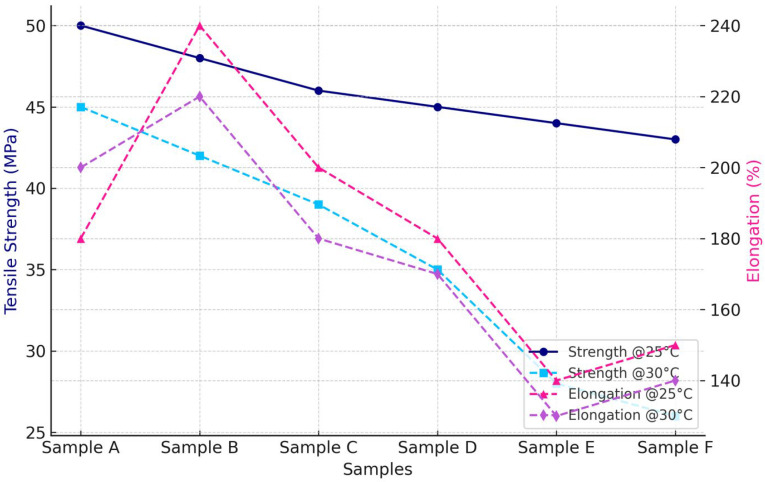
Comparison of capsule film performance under standard (25 °C/60% RH) and accelerated (30 °C/75% RH) storage conditions. Elevated temperature and humidity accelerated matrix weakening across all samples, with sharper tensile strength losses than under standard storage. Elongation increased in glycerol-rich films, but beyond Sample B, the effect reversed, suggesting network saturation and compromised structural integrity.

A comparative analysis of standard and accelerated storage conditions (Figure 5) revealed consistent reductions in tensile strength across all formulations, with greater losses observed at 30 °C/75% RH. Sample A retained the highest strength, measuring 50 MPa at 25 °C and 45 MPa at 30 °C. Sample F exhibited the lowest stability, decreasing to 43 MPa at 25 °C and 26 MPa at 30 °C.

Elongation increased progressively with glycerol content at 25 °C but followed a non-linear pattern under accelerated storage, peaking at 250% in Sample B before declining in subsequent formulations. These observations indicate that excess glycerol accelerates plasticization and weakens hydrogen bonding, whereas CHC-containing films (Samples A-C) maintained greater mechanical integrity under both storage conditions.

The elongation behavior, shown on the secondary axis, increased progressively with glycerol concentration at 25 °C, indicating greater ductility. Under heat stress (30 °C), elongation peaked at Sample B and subsequently declined, suggesting that excess glycerol saturated the polymer network and reduced structural stability. These results confirm that thermal-humidity exposure accelerates plasticization and weakens hydrogen bonding, particularly in glycerol-rich formulations. In contrast, cellulose-reinforced films (Samples A-C) retained higher mechanical stability, emphasizing the critical role of a balanced CHC–glycerol ratio in the design of storage-resilient capsule films.

Finally, under ICH-aligned storage conditions (25 °C/60% RH and 30 °C/75% RH), our samples exhibited better retention of tensile properties and fewer macroscopic defects than typically reported for neat gelatin films, which are known to be humidity-sensitive [9,10]. This aligns with literature showing that CHC-based reinforcements mitigate humidity-driven softening by restricting chain mobility and reducing free-volume changes, thereby improving stability under storage stress [9]. Collectively, these comparisons indicate that the proposed CHC–gelatin–CS system achieves a favorable balance of strength, compliance with pharmacopeial disintegration, physiologically relevant swelling, and storage stability relative to recent reports on gelatin–polysaccharide film systems.

### 3.2. Storage Appearance

#### 3.2.1. Capsule Shell Surface by Optical Microscopy

Optical micrographs showed that CHC-reinforced films exhibited more uniform surfaces and fewer visible defects than the control, particularly after conditioning at 30 °C/75% RH. The improved appearance aligns with the retention of tensile properties and pharmacopeial disintegration performance, suggesting enhanced network cohesion under storage stress. However, samples E and F had apparent air bubbles or holes. This could be because these formulations employed more glycerol than the others. Glycerol is a plasticizer, and samples E and F had apparent air bubbles or holes. This could be because these formulations employed more glycerol than the others. Glycerol is a plasticizer, and when there is more of it, it can make the film-forming solution more fluid, which can trap air when casting or drying.

#### 3.2.2. Capsule Clarity and Structural Assessment by UV–Vis and FTIR

The optical clarity of capsule films was evaluated by UV–Vis spectrophotometry at 600 nm in accordance with USP 43–NF 38 guidelines [1]. Based on the Beer–Lambert law, lower absorbance values correspond to greater transparency, as fewer particles scatter or absorb incident light [2]. As shown in Table 3, absorbance values increased progressively from 0.71 (Sample A) to 1.00 (Sample F).

In contrast, Samples D–F, representing soft-type capsules, showed higher absorbance and opacity due to thicker and more flexible matrices enriched with plasticizer [4]. These findings confirm an inverse relationship between capsule wall thickness/flexibility and optical clarity, consistent with previously observed film thickness trends. Beyond their structural implications, clarity values are also relevant as a quality attribute, influencing consumer perception and product aesthetics in pharmaceutical capsule design [5].

FTIR analysis provided molecular-level evidence of hydrogen bonding and polysaccharide–protein interactions, which directly influence capsule transparency and surface compactness. The spectra revealed characteristic O–H (~3400 cm^−1^), amide I/II (~1650/1540 cm^−1^), and C–O (~1030 cm^−1^) bands, with slight shifts and intensity changes relative to neat gelatin, indicating strengthened hydrogen bonding and electrostatic interactions. Key representative bands are summarized in Table 4, while second-derivative spectra (Supplementary Figure S1) confirmed hidden contributions in the amide and carbohydrate regions, thereby strengthening the reliability of the assignments. Beyond their structural implications, clarity values are also relevant as a quality attribute, influencing consumer perception and product aesthetics in pharmaceutical capsule design [5].

Collectively, the UV–Vis and FTIR results demonstrate that capsule clarity is controlled by both macroscopic architecture and molecular interactions: CHC-rich hard-type films exhibited thinner, smoother walls and enhanced hydrogen bonding, leading to greater transparency and stability, whereas glycerol-rich soft-type films showed thicker, more plasticized matrices with reduced clarity and weaker cohesive interactions.

#### 3.2.3. Temperature and Humidity Stability

Observations under storage conditions revealed that CHC incorporation improved surface uniformity and minimized visible defects at elevated temperature/humidity. While this does not replace instrumental thermal analysis (TGA/DSC), the combined optical and mechanical data provide relevant insight into capsule performance under practical storage stress. The temperature and humidity stability of capsule films was evaluated under controlled conditions. All six samples were stored at 25 °C and 60% RH for five days, during which no changes in odor, color, or texture were observed, indicating high stability under mild storage. In contrast, exposure to 30 °C and 75% RH resulted in noticeable softening of film texture, suggesting accelerated degradation under thermal and humidity stress [1,2].

These observations are consistent with (i) the increase in elongation and reduction in tensile strength under thermal stress [3], (ii) the presence of surface voids and structural irregularities in glycerol-rich films [4], and (iii) moisture and thickness trends, which directly influence integrity [5]. Together, the structural, mechanical, and thermal results confirm that film performance is governed by moisture level, film thickness, and glycerol content [1,4,6]. Formulations containing higher glycerol exhibited greater softness and opacity under heat, whereas lower-moisture films maintained rigidity and clarity. These findings support the applicability of CHC as a biodegradable functional ingredient, with formulation adjustments enabling application-specific tailoring of capsule properties [6,7].

### 3.3. Disintegration of Capsule Film

**Figure 6:**
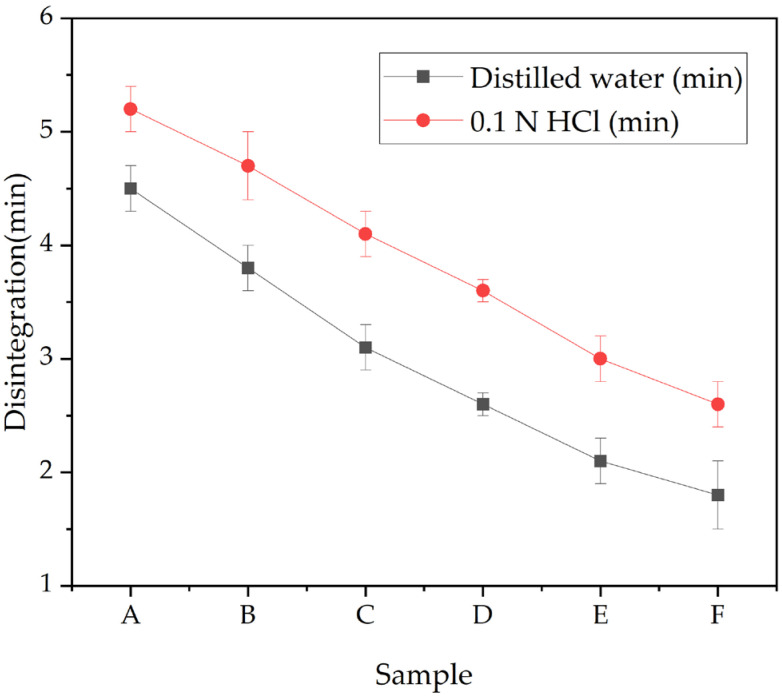
Disintegration time (mean ± SD, n = 3) of CHC-reinforced gelatin–CS capsule shells in distilled water and 0.1 N HCl at 37 °C. Tests followed the pharmacopeial procedure (basket-rack apparatus). All samples (A–F; compositions in Table 1) complied with the ≤30 min requirement in both media.

Figure 6 shows the disintegration times of the capsule films in distilled water (37 ± 0.5 °C), ranging from 4.50 min (Sample A) to 1.20 min (Sample F). All formulations disintegrated far below the pharmacopeial limit of 30 min required by USP and EP standards [13,14], confirming their suitability for oral dosage applications.

Comparative testing in acidic (0.1 N HCl) and neutral (distilled water) media further revealed that disintegration proceeded more rapidly under acidic conditions, reflecting the increased solubility of gelatin at low pH. Compositional differences also influenced the disintegration rate: films with higher glycerol and lower cellulose contents (e.g., Sample F) exhibited the fastest disintegration due to enhanced hydrophilicity, swelling capacity, and matrix loosening. Conversely, CHC-rich formulations showed slightly longer but still pharmacopeia-compliant disintegration times, consistent with their more compact and rigid network structure.

**Figure 7:**
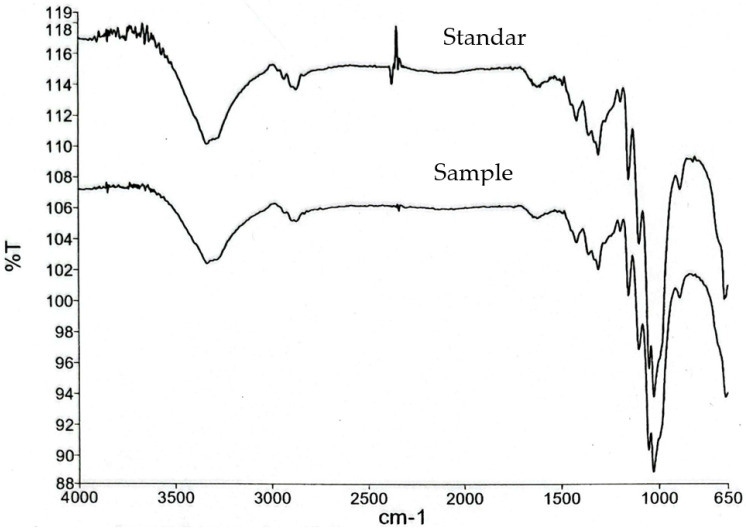
FTIR spectra (absorbance) of CHC-reinforced gelatin–CS films vs. standard, baseline-corrected, and normalized to amide I. Annotated bands indicate hydrogen-bond-mediated interactions, supporting matrix compatibility. FTIR spectra of the samples (wavenumber in cm^−1^).

**Figure 8:**
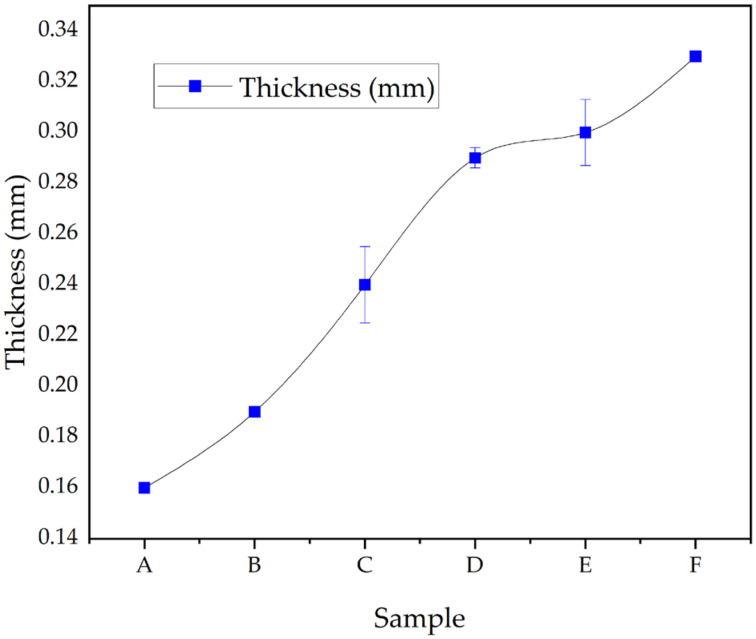
Thickness of CHC-reinforced gelatin–CS capsule films (Samples A–F) measured after drying (mean ± SD, n = 3). For each replicate, the thickness was averaged from five random positions per strip. Thickness generally increases from A to F, consistent with higher glycerol and CHC contents in the formulations (see Table 1).

**Figure 9:**
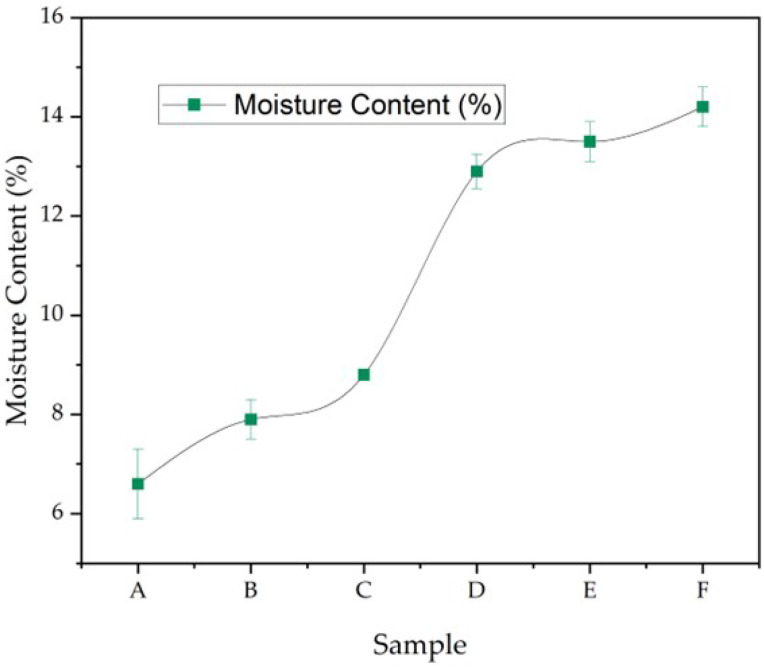
Moisture content of Samples A–F (mean ± SD, n = 3), determined by gravimetric drying to constant weight; the rise from A→F follows increasing glycerol/CHC loading (Table 1).

Regarding pharmacopoeial performance, our films/capsules met the ≤30 min disintegration limit in both water and 0.1 N HCl, comparable to recent gelatin-based systems reinforced with polysaccharides or nano-cellulosic fillers [5,6]. Notably, several prior studies reported slower disintegration when high filler fractions induced densified networks; by contrast, our CHC levels maintained compliance without sacrificing mechanical integrity, which we attribute to balanced plasticization (glycerol) and the compatible CHC–gelatin–CS network [5]. Figure 7 and Figure 8 show the mechanical–structural relationships… These findings align with the moisture content results (Figure 9), where soft-type formulations exhibited higher water uptake and faster breakdown. Overall, the data emphasize that capsule composition, particularly the glycerol–CHC ratio, governs disintegration kinetics and thereby influences drug release behavior. Such tunability is advantageous for tailoring capsules toward specific therapeutic targets, such as rapid gastric release versus more controlled intestinal delivery. In both test media, all capsule formulations demonstrated satisfactory disintegration. Notably, the disintegration rate was faster in acidic medium (0.1 N HCl) than in neutral water, attributed to enhanced gelatin solubility at lower pH. Additionally, formulations containing higher glycerol and lower CHC concentrations (e.g., Sample F) exhibited more rapid disintegration, likely due to increased hydrophilicity, swelling, and matrix loosening.

These findings confirm that the inclusion of natural hydrophilic additives such as glycerol and CHC not only improves moisture retention but also facilitates rapid water uptake and matrix disintegration. As such, capsule composition plays a pivotal role in determining the release kinetics of active pharmaceutical ingredients (APIs), aligning with specific therapeutic targets, such as gastric or intestinal drug release.

### 3.4. FTIR Analysis of Gelatin–CHC Composite Films

The FTIR spectra of the capsule films (Figure 7) revealed characteristic peaks associated with gelatin, cellulose, and starch. A broad absorption band around 3400 cm^−1^ was assigned to O–H stretching vibrations, reflecting extensive hydrogen bonding among hydroxyl groups. The band near 2900 cm^−1^ corresponded to C–H stretching of aliphatic chains. Distinct absorptions at ~1650 cm^−1^ (amide I) and ~1540 cm^−1^ (amide II) confirmed the peptide backbone of gelatin, while the peak at ~1030 cm^−1^ was attributed to C–O stretching from polysaccharide units of CHC and CS.

Compared with the standard gelatin spectrum, the composite films exhibited slight shifts and variations in intensity, particularly in the amide I/II and C–O stretching regions. These spectral changes indicate intermolecular hydrogen bonding and electrostatic interactions between gelatin and CHC. The broadening of the O–H band and the enhanced C–O signal further suggest successful incorporation of CHC into the gelatin–CS network. Such molecular-level interactions are expected to restrict chain mobility, thereby enhancing film cohesion and stability. This finding is consistent with the improved tensile strength and thermal resistance observed in CHC-reinforced formulations.

Overall, the FTIR analysis confirms effective blending and compatibility of the biopolymer components within the capsule matrix. The FTIR spectra of the composite films showed a broad O–H band (~3400 cm^−1^), amide I/II bands (~1650/1540 cm^−1^), and a pronounced C–O band (~1030 cm^−1^), consistent with gelatin–CHC–CS systems. Relative to neat gelatin, slight shifts and intensity changes in amide I/II and C–O regions indicate strengthened hydrogen-bonding and possible electrostatic interactions between the peptide backbone and cellulose hydroxyls. This pattern mirrors prior observations in gelatin–polysaccharide blends, where the incorporation of CHC or CS perturbs the local environment of amide groups and increases intermolecular cohesion. In our films, the broadening of the O–H band together with the enhanced C–O signal supports successful CHC integration into the gelatin–CS network.

These molecular-level interactions rationalize the macroscopic trends observed. First, mechanical behavior: CHC-rich formulations (Samples A–C) retained higher tensile strength and resisted softening during storage, consistent with reduced chain mobility imparted by interfacial H-bonding. In contrast, glycerol-rich films (Samples D–F) showed larger elongation but lower strength, reflecting plasticization and increased free volume that weaken cohesive forces. Second, moisture and swelling: higher glycerol content increased water uptake and swelling, which facilitates disintegration but also accelerates plasticization under heat–humidity stress. CHC moderated this effect by promoting a denser, more hydrogen-bonded network that limits excessive hydration. Third, morphology and clarity: optical microscopy revealed microvoids/bubbles in glycerol-rich matrices that correlate with higher turbidity (A600) and lower strength; smoother, denser surfaces in CHC-rich films align with better clarity and mechanical stability. Finally, temperature and humidity accelerated storage at 30 °C/75% RH amplified these contrasts: CHC-reinforced films maintained integrity longer, whereas over-plasticized films reached a ductility plateau and lost resilience. Collectively, the data support a composition–structure–property framework in which the glycerol–CHC balance governs hydrogen-bond density, water sorption, microstructure (voids vs. compactness), and, ultimately, mechanical and disintegration performance. This tunability is attractive for tailoring capsules toward rapid gastric release (higher glycerol, thinner films) versus more storage-resilient shells (higher CHC, controlled moisture, moderate thickness).

Key representative bands are summarized in Table 5, while second-derivative spectra (Supplementary Figure S1) further confirm hidden contributions in the amide and carbohydrate regions, supporting the reliability of these assignments.

### 3.5. Capsule Film Thickness and Moisture Content

#### 3.5.1. Capsule Film Thickness

Capsule thickness is a critical parameter, as it influences mechanical strength, moisture retention, and disintegration time. While thicker films may offer improved durability and moisture barrier properties, excessive thickness can delay disintegration and increase material cost and production time. Figure 8 shows the variation in film thickness across six capsule samples, ranging from 0.16 mm (Sample A) to 0.33 mm (Sample F). The gradual increase in thickness reflects the influence of formulation variables—particularly the amounts of CHC and glycerol, which contribute to the viscosity and density of the gel matrix during film casting and drying.

The rise in CHC content is likely to increase the viscosity of the film-forming solution, resulting in thicker films upon drying. Similarly, glycerol contributes to the swelling and volumetric expansion of the polymer matrix, further affecting thickness. These formulation-driven changes have direct implications for capsule functionality [15].

According to pharmacopeial standards, hard capsule films generally fall within the 0.15–0.25 mm range, while soft capsule films are typically 0.25–0.35 mm [1,2,13]. Based on these classifications, Samples A–C fall within the acceptable range for hard capsules, while Samples D–F align with the criteria for soft capsules.

Achieving the right balance in film thickness is essential to ensure both structural stability and efficient drug release. Optimized thickness contributes to product integrity, protection of active pharmaceutical ingredients, and predictable gastrointestinal performance.

#### 3.5.2. Moisture Content

Figure 9 presents the moisture content of six capsule shell samples, showing a gradual increase from Sample A to Sample F, with values of 6.6%, 7.9%, 8.8%, 12.9%, 13.5%, and 14.0%, respectively. According to pharmacopeial standards, the acceptable moisture content is 6–12% for hard capsules and 12–20% for soft capsules, which ensures their physical integrity during storage and handling [11,16].

Based on these standards, Samples A–C fit within the acceptable range for hard capsules, while Samples D–F exceed 12%, aligning with the moisture profile of soft capsule formulations. Adequate moisture content is critical to prevent brittleness or cracking during storage, especially in gelatin-based systems [10]. For soft capsules, plasticizers such as glycerol play a key role in enhancing flexibility by retaining moisture [4,8]. The observed trend suggests that formulation variables, particularly glycerol and CHC content, directly influence moisture retention and capsule classification. Consequently, precise control of moisture content becomes a crucial design factor for ensuring mechanical performance, storage stability, and overall capsule functionality in pharmaceutical applications [9,10,11].

This classification is consistent with formulation composition. Increasing glycerol concentration elevated water retention due to its hygroscopic nature, while higher CHC content reduced water uptake by providing a more rigid, less hydrophilic structure. These opposing effects highlight the critical balance between plasticizer and reinforcement in determining capsule moisture behavior. The observed trend also correlates with functional properties: higher-moisture films are expected to exhibit greater flexibility but reduced tensile strength, whereas lower-moisture films may be mechanically stronger but more brittle. Maintaining moisture content within the optimal range is therefore essential to prevent cracking, brittleness, or excessive softening, directly impacting capsule durability and handling stability.

Overall, moisture control emerges as a key formulation parameter that not only dictates capsule classification (hard vs. soft) but also influences mechanical performance and storage stability, underscoring the importance of precise adjustment of glycerol–CHC ratios in capsule design [9,10,11].

### 3.6. Swelling Index

**Figure 10:**
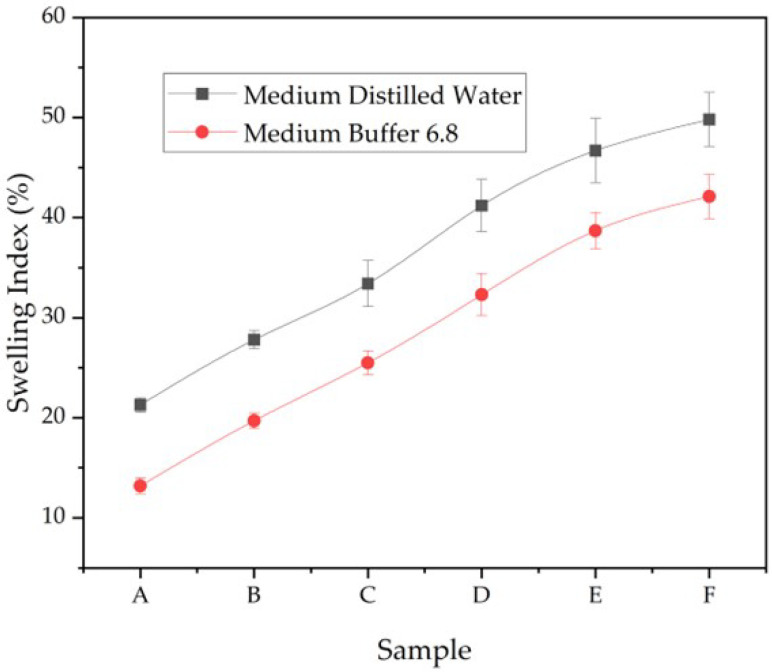
Swelling index (%) of CHC-reinforced gelatin–CS capsule films (Samples A–F) in distilled water and phosphate buffer (pH 6.8) at 37 °C (mean ± SD, n = 3). Swelling is higher in water than in buffer, consistent with ionic screening in the buffered medium; the increase from A→F follows higher glycerol/CHC contents (Table 1).

The swelling index quantifies the capsule film’s capacity to absorb fluid, reflecting matrix hydration and integrity under physiological conditions. Pre-weighed dried samples were immersed in distilled water and a pH 6.8 buffer at 37 °C for 30 min, and the relative weight gain was expressed as swelling index (%). As shown in Figure 10, all formulations exhibited higher swelling in distilled water compared to phosphate buffer, indicating that hydration was more pronounced under neutral aqueous conditions than in buffered medium.

The swelling trends correlate with the disintegration behavior (Figure 6), where highly swollen films also disintegrated more rapidly due to accelerated water uptake and network loosening. Excessive swelling, however, could compromise mechanical stability, whereas moderate swelling supports balanced hydration and integrity. For swelling in PBS, our profiles are consistent with hydrophilic gelatin–polysaccharide matrices that exhibit >100% mass uptake at early times before approaching equilibrium due to ionic screening in physiological saline [7,8]. Compared with related systems, our equilibrium swelling remained within a range supportive of capsule handling while avoiding excessive gelation/softening that could compromise integrity during processing [7].

Overall, these findings demonstrate that the glycerol–CHC ratio governs capsule swelling behavior, providing a means to tailor hydration, disintegration, and ultimately drug release kinetics for site-specific delivery in the gastrointestinal tract.

### 3.7. Ash Content

**Figure 11:**
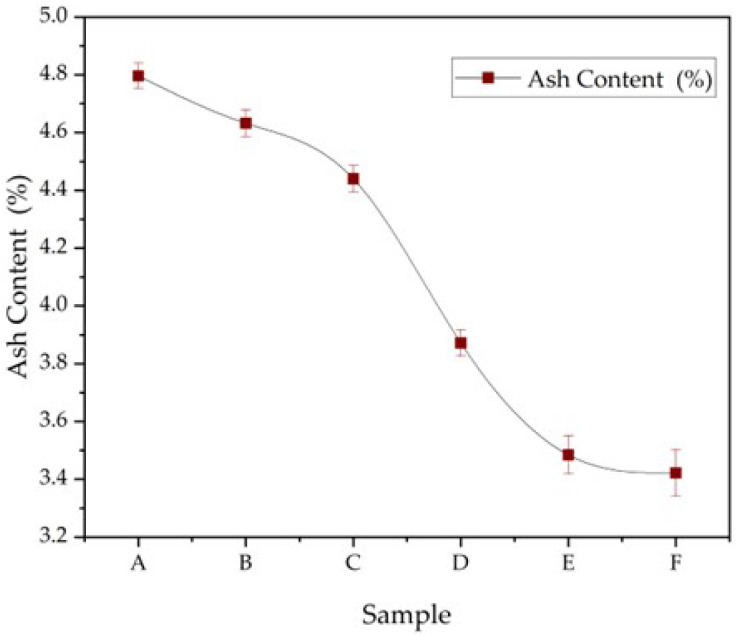
Ash content (%) of CHC-reinforced gelatin–CS capsule films (Samples A–F) (mean ± SD, n = 3). Ash was determined gravimetrically as the inorganic residue after incineration in a muffle furnace to constant weight (see Methods). The decreasing trend from A to F is consistent with the lower CHC loading in later formulations (Table 1).

Figure 11 shows the ash content of capsule films following incineration at 600 °C in a muffle furnace. The values decreased from 4.80% in Sample A to 3.42% in Sample F, indicating a reduction in inorganic residue across the formulations. According to the Indonesian Ministry of Health (1995) [16], capsule shells should contain less than 5% ash. All formulations in this study complied with this requirement, confirming low levels of inorganic or non-combustible materials such as mineral fillers or residual processing agents [1].

Ash content is a critical parameter for assessing purity and quality in medicinal products. Elevated ash levels may reflect contamination, excessive use of inorganic excipients, or insufficient removal of residues during processing. The lower ash values observed in Samples D to F suggest higher purity, likely due to the greater incorporation of natural polymers such as cellulose and reduced reliance on inorganic additives.

## 4. Conclusions

This study demonstrates the feasibility of CHC as a sustainable and biodegradable reinforcement material for gelatin-based capsule shells. The composite films prepared from CHC, CS, gelatin, and glycerol exhibited desirable properties, including mechanical strength, flexibility, controlled disintegration, and biodegradability.

Under standard storage conditions (25 °C/60% RH), the films maintained their physical integrity without changes in color, odor, or texture. Accelerated conditions (30 °C/75% RH) induced softening and a marked reduction in tensile strength, underscoring the importance of thermal–humidity stability testing in capsule design. These results confirm that CHC incorporation enhances structural integrity, while excessive plasticizer compromises stability under stress.

The incorporation of CHC not only improves capsule durability and functionality but also contributes to environmentally friendly and halal-compliant alternatives for pharmaceutical and nutraceutical applications. An optimal compositional window was identified—CS (10–30%), cellulose (10–55%), gelatin (15–35%), and glycerol (5–45%)—that balances strength, flexibility, and biodegradability. This formulation framework provides a basis for developing next-generation capsule systems tailored for long-term drug delivery and sustainable biomedical applications.

## Figures and Tables

**Table 1:** Composition of Capsule Film Formulations.

Sample	Gelatin (g)	CS (g)	CHC (g)	Glycerol (g)
A	30	10	55	4.75
B	25	15	45	12.75
C	35	10	35	16
D	25	20	30	18.75
E	20	25	20	22.75
F	15	30	10	24.75

**Table 2:** Mechanical properties and storage appearance of capsule films during five days.

Sample	Tensile Strength @25 °C (MPa)	Elongation @25 °C (%)	Tensile Strength @30 °C (MPa)	Elongation @30 °C (%)	Mechanical Classification
A	50.2 ± 1.4	66.7 ± 3.2	45.1 ± 1.1	183.3± 3.1	Excellent (rigid, elastic)
B	48.7 ± 1.6	83.3 ± 3.9	42.3 ± 1.3	250.1 ± 3.5	Flexible, stable
C	47.3 ± 1.8	95.2 ± 4.1	39.3 ± 1.2	171.4 ± 3.9	Moderate
D	45.9 ± 1.5	120.8 ± 5.0	35.3± 1.7	160.9± 4.8	Ductile
E	44.5 ± 1.7	137.2 ± 4.3	28.2 ± 1.5	127.3± 3.7	Soft, reduced integrity
F	43.1 ± 1.7	153.8 ± 4.6	26.7± 1.7	140 ± 4.2	Poor mechanical retention

Values are reported as mean ± SD (n = 3); tensile testing was conducted according to ASTM D882. Storage conditions follow ICH stability guidance: 25 °C/60% RH (long-term) and 30 °C/75% RH (accelerated).

**Table 3:** The Optical Microscopy of Capsule Films: Samples A through F.

Sample	Visual/Microscopic Surface Observation	Interpretation
A–B	Smooth, dense surface; no visible pores or defects under 100× microscopy	Well-formed matrix with good film integrity correlates with high tensile strength and low ductility
C–D	Slightly rough surface; occasional shallow voids or minor irregularities	Moderate matrix disruption; corresponds with balanced strength and flexibility
E–F	Irregular surface with visible bubbles and microvoids under light microscopy and SEM	Poor film formation with trapped air or moisture explains low strength and high elongation

**Table 4:** FTIR-Based Clarity Assessment of Hard and Soft Capsule Samples.

Sample	Absorbance (cm^−1^)	Interpretation
A	0.7109	Clearer (hard capsule)
B	0.7499	–
C	0.8005	–
D	0.8498	–
E	0.9724	–
F	1.0023	Less clear (soft capsule)

**Table 5:** Key FTIR Absorption Bands of Gelatin–CHC Composite Films.

Wavenumber (cm^−1^)	Assignment	Interpretation
~3400	O–H stretching	Hydrogen bonding (gelatin–cellulose)
~2900	C–H stretching	Aliphatic chains
~1650	Amide I (C=O stretch)	Peptide backbone of gelatin
~1540	Amide II (N–H bend)	Gelatin–cellulose interaction
~1030	C–O stretching	Polysaccharide (cellulose/starch) units

## Data Availability

The original contributions presented in this study are included in the article. Further inquiries can be directed to the corresponding author.

## Supplementary Materials

The following supporting information can be downloaded at: https://www.mdpi.com/article/10.3390/polym17202803/s1, Figure S1: ATR-FTIR absorbance spectra of gelatin–CHC composite films with second-derivative overlay (red). The second-derivative analysis reveals hidden or overlapping peaks, particularly in the amide I/II and C–O–C regions, thereby confirming the band assignments summarized in Table 5.
