# Supplementary material for: Structural and Functional Enhancement of Halal Gelatin Capsules Reinforced with Corn Husk Cellulose
Source: Polymers (Basel). 2025 Oct 21;17(20):2803. doi: 10.3390/polym17202803 (PMC12567270; doi:10.3390/polym17202803)
Supplement: Supplementary file 1 [file polymers-17-02803-s001.zip › polymers-3868337-SI.pdf]

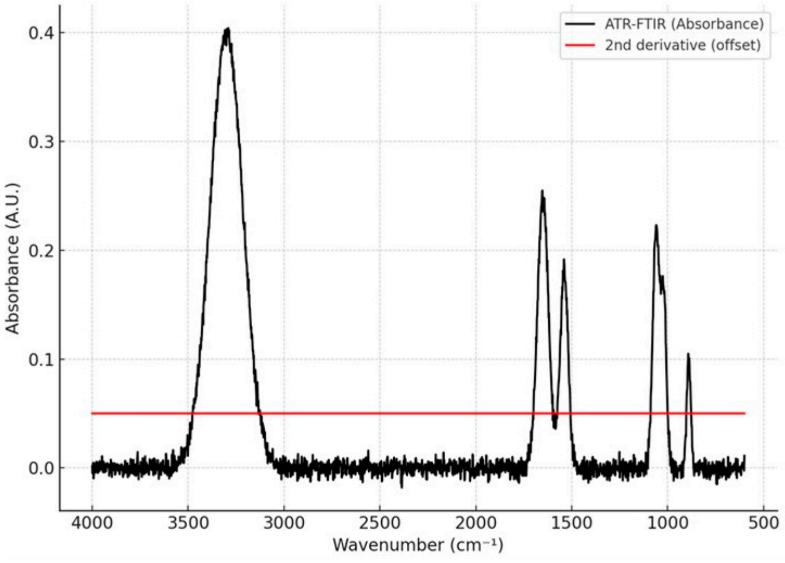

Figure S1. ATR-FTIR absorbance spectra of gelatin–CHC composite films with second-derivative overlay (red). The second-derivative analysis reveals hidden or overlapping peaks, particularly in the amide I/II and C–O–C regions, thereby confirming the band assignments summarized in Table 5.
